# Supplementary material for: Rapid and Efficient Enrichment of Snake Venoms from Human Plasma Using a Strong Cation Exchange Tip Column to Improve Snakebite Diagnosis
Source: Toxins (Basel). 2021 Feb 13;13(2):140. doi: 10.3390/toxins13020140 (PMC7917991; doi:10.3390/toxins13020140)
Supplement: Supplementary file 1 [file toxins-13-00140-s001.pdf]

Supplementary Materials: Rapid and Efficient Enrichment of Snake Venoms from Human Plasma Using a Strong Cation Exchange Tip Column to Improve Snakebite Diagnosis

Chien-Chun Liu, Ya-Han Yang, Yung-Chin Hsiao, Po-Jung Wang, Jo-Chuan Liu, Chien-Hsin Liu, Wen-Chin Hsieh, Chih-Chuan Lin and Jau-Song Yu

Table S1. The pI values of human plasma abundant proteins (Top-14) and snake venom proteins.

Species	Accession Number	Protein Name	pI Value (calculated)
Human specimen	P02768	Human serum albumin	5.92
	-	IgG	6.6-7.2
	-	IgM	-
	-	IgA	4.6-6.8
	P00738	Haptoglobin	6.13
	P02787	Transferrin	6.18
	P01009	Alpha1-antitrypsin	5.37
	P01023	Alpha2-macroglobulin	6.03
	P01024	Complement C3	6.02
	P02763	Alpha1-acid glycoprotein	4.93
	P02647	Apolipoprotein AI	5.56
	P02766	Transthyretin	5.49
	P02652	Apolipoprotein AII	6.27
	P04114	Apolipoprotein B-100	6.58
N. atra	A4FS04	Acidic phospholipase A2 natratoxin	4.9
	A8QL59	Zinc metalloproteinase-disintegrin-like NaMP	5.07
	P00598	Acidic phospholipase A2 1	5.17
	Q91133	Acidic phospholipase A2 2	5.52
	P61898	Venom nerve growth factor	6.08
	D6PXE8	Zinc metalloproteinase-disintegrin-like atrase-B	7.28
	D5LMJ3	Zinc metalloproteinase-disintegrin-like atrase-A	7.66
	D3TTC1	Zinc metalloproteinase-disintegrin-like kaouthiagin-like	7.68
	Q7T1K6	Cysteine-rich venom protein natrin-1	8.3
	Q5ZPJ7	Kunitz-type serine protease inhibitor	8.32
	Q9DEQ3	Neurotoxin homolog NL1	8.46
	P80958	Cobrotoxin-b	8.5
	P60770	Cobrotoxin	8.51
	D3TTC2	Zinc metalloproteinase-disintegrin-like atragin	8.7
	Q9W717	Neurotoxin-like protein NTL2	8.71
	Q9YGI1	Probable weak neurotoxin NNAM3	8.86
	Q9YGI2	Probable weak neurotoxin NNAM1	8.99
	O93422	Long neurotoxin homolog	8.99
	Q98956	Cytotoxin 1b	9.02
	Q91135	Cytotoxin I-like P-15	9.03
	Q98958	Cytotoxin 1d/1e	9.03
	Q7ZZN8	Cysteine-rich venom protein natrin-2	9.07
	Q9YGI4	Probable weak neurotoxin NNAM2	9.07
	Q9W6W6	Cytotoxin 10	9.15
	P60304	Cytotoxin 1	9.16
	Q91136	Cytotoxin I-like T-15	9.16
	Q98957	Cytotoxin 1a	9.16
	P79810	Cytotoxin 1c	9.16
	P60814	Probable weak neurotoxin NNAM2I	9.21
	P01442	Cytotoxin 2	9.28
	P80245	Cytotoxin 6	9.28
	P49123	Cytotoxin 8	9.28
	Q98965	Cytotoxin 6	9.3

	Q8UUK0	Cytotoxin homolog Clbp-3	9.33
	P62375	Cytotoxin A5	9.36
	Q91126	Cardiotoxin 7a	9.36
	P60308	Cytotoxin SP15c	9.36
	Q91996	Cardiotoxin 7''	9.36
	Q91137	Cytotoxin homolog 5	9.36
	P60301	Cytotoxin 3	9.38
	P07525	Cytotoxin 5	9.38
	Q98960	Cytotoxin 3b	9.38
	Q98961	Cytotoxin 5	9.38
	Q9W716	Cytotoxin homolog 5V	9.38
	Q98959	Cytotoxin 3a	9.38
	P01443	Cytotoxin 4	9.41
	Q91124	Cytotoxin 8	9.41
	Q98962	Cytotoxin 3d	9.41
	Q9W6W9	Cytotoxin 4N	9.41
	P49122	Cytotoxin 7	9.44
	P60306	Cytotoxin SP13b	9.48
	P60307	Cytotoxin SP15a	9.48
	P60309	Cytotoxin SP15d	9.59
	D9IX97	Natriuretic peptide Na-NP	9.7
	B6S2X0	Cathelicidin-related peptide Na_CRAMP	11.59
B. multicinctus	P17934	Acidic phospholipase A2 beta-bungarotoxin A4 chain	5.26
	P00619	Acidic phospholipase A2 beta-bungarotoxin A3 chain	6.1
	P15816	Kappa-2-bungarotoxin	6.69
	P00606	Acidic phospholipase A2	6.82
	Q90251	Acidic phospholipase A2 beta-bungarotoxin A6 chain	6.93
	P00617	Basic phospholipase A2 beta-bungarotoxin A1 chain	7.57
	O12962	Kappa-5-bungarotoxin	7.64
	A1XXJ9	C-type lectin BML-2	7.68
	Q9PW19	Cardiotoxin-like protein BMLCL	7.7
	Q9PTA5	Basic phospholipase A2 beta-bungarotoxin A-AL4 chain	7.94
	P59018	Basic phospholipase A2 beta-bungarotoxin A5 chain	7.97
	Q9PU97	Basic phospholipase A2 beta-bungarotoxin A7 chain	7.97
	Q9PTA7	Basic phospholipase A2 beta-bungarotoxin A-AL2 chain	7.98
	Q9PTA1	Basic phospholipase A2 beta-bungarotoxin A-AL1 chain	8
	P01398	Kappa-bungarotoxin	8.12
	O12961	Kappa-4-bungarotoxin	8.12
	Q9YGH0	Cytotoxin-like protein TA-BMBGT3	8.12
	Q9YGI0	Short neurotoxin homolog NTL1	8.18
	Q7ZT13	Neurotoxin-like protein pMD18-NTL1/2/4/5	8.18
	Q800Y3	Neurotoxin-like protein pMD18-NTL3	8.18
	Q9PTA6	Basic phospholipase A2 beta-bungarotoxin A-AL3 chain	8.21
	Q1RPS8	Kunitz-type serine protease inhibitor homolog beta-bungarotoxin B6 chain	8.23
	P34128	Venom nerve growth factor	8.31
	P15818	Long neurotoxin homolog	8.36
	O12963	Long neurotoxin homolog TA-bm16	8.37
	P60615	Alpha-bungarotoxin isoform A31	8.38
	P60616	Alpha-bungarotoxin isoform V31	8.38
	P00618	Basic phospholipase A2 beta-bungarotoxin A2 chain	8.41
	Q90WI6	C-type lectin BML-1	8.41
	P15817	Kappa-3-bungarotoxin	8.42
	Q9W727	Muscarinic toxin-like protein	8.46
	A8QL51	L-amino-acid oxidase	8.49
	Q8JFX7	Muscarinic toxin BM14	8.57
	Q70WS8	Neurotoxin BM10-1-like	8.57
	Q9YGH9	Long neurotoxin homolog NTL2	8.58
	Q9YGJ0	Gamma-bungarotoxin	8.59
	Q9W729	Kappa-6-bungarotoxin	8.61
	P79688	Cardiotoxin homolog TA-ctx-like	8.69
	Q0PL65	Kunitz-type serine protease inhibitor homolog beta-bungarotoxin B5-B chain	8.96
	B4ESA2	Kunitz-type serine protease inhibitor PILP-1	9.04
	P43445	Short neurotoxin homolog	9.05
	B4ESA3	Kunitz-type serine protease inhibitor PILP-2	9.1

Q1RPT0	Kunitz-type serine protease inhibitor homolog beta-bungarotoxin B4 chain	9.1
Q9W728	Kunitz-type serine protease inhibitor homolog beta-bungarotoxin B3 chain	9.12
Q1RPS9	Kunitz-type serine protease inhibitor homolog beta-bungarotoxin B5 chain	9.12
P00989	Kunitz-type serine protease inhibitor homolog beta-bungarotoxin B2 chain	9.25
P00987	Kunitz-type serine protease inhibitor homolog beta-bungarotoxin B1 chain, major component	9.28
A8QL49	Zinc metalloproteinase-disintegrin-like BmMP	9.37
Q9YGI8	Short neurotoxin homolog NTL4	9.45
B4ESA4	Kunitz-type serine protease inhibitor PILP-3	9.63
P0DMD5	Natriuretic peptide BM026	10.08

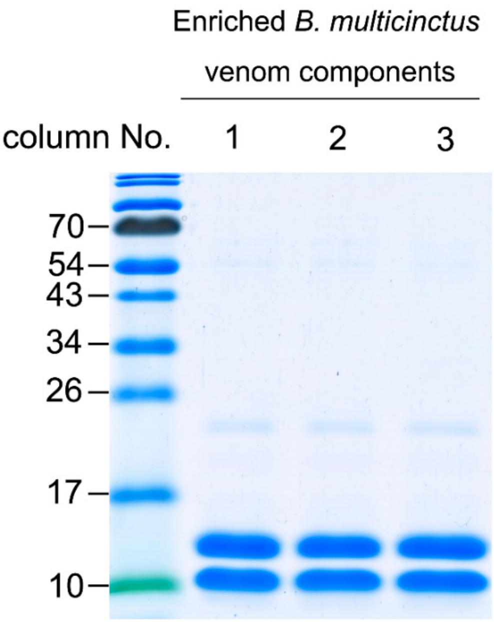

Figure S1. Reproducibility of *B. multicinctus* venom enrichment using different manual SCX-tip columns. *B. multicinctus* venom (20 μg protein) was subjected to venom protein enrichment using three different SCX tip columns. Venom proteins absorbed on each column were eluted by 300 mM NaCl and analyzed by SDS-PAGE, followed by Coomassie blue staining.

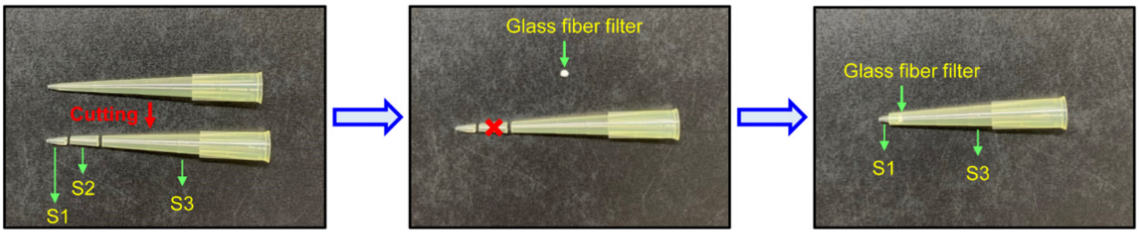

Figure S2. Procedure of making a tip column. A 200 μL polypropylene tip was cut into 3 segments. Segment-1 (S1) was placed onto Segment-3 (S3), and pieces of glass fiber filter were inserted into the upper site of S1 to form the tip column.
